# Predictive Modeling of the Progression of Alzheimer’s Disease with Recurrent Neural Networks

**DOI:** 10.1038/s41598-018-27337-w

**Published:** 2018-06-15

**Authors:** Tingyan Wang, Robin G. Qiu, Ming Yu

**Affiliations:** 1 Health Care Services Research Center, Department of Industrial Engineering, Tsinghua University, Beijing, 100084 China; 2 Big Data Lab, Division of Engineering and Information Science, The Pennsylvania State University, Malvern, PA 19355 USA

## Abstract

The number of service visits of Alzheimer’s disease (AD) patients is different from each other and their visit time intervals are non-uniform. Although the literature has revealed many approaches in disease progression modeling, they fail to leverage these time-relevant part of patients’ medical records in predicting disease’s future status. This paper investigates how to predict the AD progression for a patient’s next medical visit through leveraging heterogeneous medical data. Data provided by the National Alzheimer’s Coordinating Center includes 5432 patients with probable AD from August 31, 2005 to May 25, 2017. Long short-term memory recurrent neural networks (RNN) are adopted. The approach relies on an enhanced “many-to-one” RNN architecture to support the shift of time steps. Hence, the approach can deal with patients’ various numbers of visits and uneven time intervals. The results show that the proposed approach can be utilized to predict patients’ AD progressions on their next visits with over 99% accuracy, significantly outperforming classic baseline methods. This study confirms that RNN can effectively solve the AD progression prediction problem by fully leveraging the inherent temporal and medical patterns derived from patients’ historical visits. More promisingly, the approach can be customarily applied to other chronic disease progression problems.

## Introduction

### Significance of predicting dementia progression

As of 2017, approximately 5.4 million Americans in the US live with Alzheimer’s disease (AD), which is the most common form of dementia. According to the US National Alzheimer’s Coordinating Center (NACC), AD is one of leading causes of death in the US. Moreover, for a patient with AD, his or her AD condition will chronically and progressively deteriorate over a long period of time. However, as of April of 2018 there exists no effective cure for AD. In other words, AD cannot be reversed or cured with today’s medicines and treatments. Unless a method of prevention or treatment will be discovered, the estimated total cost of care of people with Alzheimer’s and other dementias in the US will grow to about $1 trillion in 2050 from an estimated $226 billion in 2015^1^. It is known that the social and psychological burden on individuals and families will be even more daunting than the costs of care.

While waiting for significant progress of developing AD cure medicines, many researchers have been looking for alternative, viable, and cost-effective solutions that help fill in the gap of the needed care and treatment for AD patients^2–8^. A very promising approach has been widely explored, focusing on early prediction and positive intervention at the personalized and comfortable level, which inherently and truly varies with patients and keeps changing over time. An appropriate and positive intervention includes ways of facilitating AD patients with right and effective levels of lifestyle changes and brain training. Therefore, understanding and predicting how AD develops on an individual patient basis over time is the key to the success of enabling early intervention of AD and accordingly providing personalized healthcare services in an effective manner^1,2^.

### Studies relevant to modeling disease progression

Traditional time series methods and machine learning algorithms have been widely applied to AD progression modeling and severity classification problems. Sukkar *et al*.^3^ applied hidden Markov chains to model AD progression; Zhou *et al*.^4^ proposed a convex fused sparse group Lasso formulation to predict AD patients’ cognitive scores at different time points; Zhou *et al*.^5^ then used a multi-task regression model to predict Mini Mental State Examination score and AD Assessment Scale Cognitive subscale score; Liu *et al*.^6^ identified the transitional patterns of AD using a series of joint random-effects transition models; Huang *et al*.^7^ proposed a nonlinear regression-based random forest model to predict longitudinal AD clinical scores; Gavidia-Bovadilla *et al*.^8^ proposed aging-based null models for early diagnosis of AD; Hou *et al*.^9^ proposed a model to predict AD longitudinal scores by estimating clinical scores from MRI data at multiple time points; O’Kelly^10^ used supervised machine learning algorithms to classify the statuses of AD patients; Qiu *et al*.^2^ applied decision tree algorithms to classify the progression statuses of AD patients. Note that many researchers have also focused on other diseases progression modeling^11–19^.

Although the above-mentioned researchers have made promising progress in studies related to diseases progression modeling^2–19^, many modeling challenging issues still remain^20^. For example, it is difficult for traditional time series methods to handle high-dimensional longitudinal data. Rather than predicting disease’s future status, the literature largely focuses on disease progression modeling using hidden Markov models and multi-task regression models, which predict the progression statuses of diseases at known time points based on the collected information relevant to those time points, or explores classifying progression stages only within a narrow observation window^2–19^.

Unlike the problem of disease progression modeling covered by the existing literature, we aim to use AD patients’ medical information at historical time points to predict their disease progression stages at a future time point. In general, it is extremely challenging to capture and derive temporal patterns to help predict accurately the future progressions of AD patients due to the fact that AD patients’ data are highly heterogeneous^2,21^. Indeed, regarding longitudinal electronic health records (EHR) describing the length of progression staging and the progressive rate at different AD stages over the years, each patient’ EHR is unique^22^. Moreover, time intervals between two consecutive visits are often irregular or uneven. However, for the future progression prediction problem under study, traditional machine learning algorithms predict diseases’ staging simply by aggregating longitudinal features rather than leveraging their longitudinal temporal patterns. As a result, modeling accuracies suffer to some extent. Therefore, it is necessary to explore a new approach for the future disease progression predictive problems.

### Predictive modeling with RNN in healthcare

RNN is naturally good at capturing longitudinal temporal patterns^23,24^. Recently, RNN models have shown great potential in healthcare applications: predicting the diagnosis and medications of the subsequent visit for a patient with gated recurrent units (GRU) RNN^25^, predicting kidney transplantation endpoints within the future six or twelve months using different RNN variants^26^, early detecting heart failure onsets using GRU models^27^, predicting the onsets of multiple conditions using Long Short-Term Memory (LSTM) models^28^, classifying diagnoses for pediatric intensive care unit (PICU) patients using LSTM networks^29^, predicting PICU’s mortality with LSTM networks^30^, predicting risk of mortality, physiologic decline, length of stay, and phenotype simultaneously with a Multitask LSTM model^31^, learning patients’ similarities for patients with Parkinson’s disease using a 2-D GRU model^32^. These studies applied various RNN models for medical prediction tasks through effectively leveraging the temporal relations among longitudinal patient data. As of April of 2018, however, RNN has not been well adopted in the AD future progression predictive modeling.

## Objective

To address this above-mentioned interesting, challenging, while long-overdue problem, we investigate whether RNN can predict the future progression of AD through fully leveraging inherent longitudinal temporal information of patients’ historical visits. Particularly, we aim to predict the AD progression stage of the next hospital visit in the future for a patient only based on the information of his/her historical visits. The Global Staging Clinical Dementia Rating (CDR) score is a clinical comprehensive metric to assess the dementia levels, which has been well adopted to define Dementia (including AD) progression stages^7,33^. Note that the Global CDR score is derived from the rating scores of six cognitive elements defined as the standard CDR scale according to clinical scoring rules. The standard CDR scale was primarily developed by Washington University School of Medicine for staging the dementia severity of AD^34^. A patient who is clinically diagnosed with dementia can be at five different stages with respect to the Global CDR score: 0 (no impairment), 0.5 (questionable impairment), 1 (mild impairment), 2 (moderate impairment), and 3 (severe impairment)^34^. Hence, the Global CDR score (i.e., CDRGLOB define in the NACC database) is utilized to define the AD progression stages in this study. Note that the proposed model is neutral to etiologic diagnoses, which implies that other metrics defining AD progression stages can be used to replace the Global CDR score if necessary.

## Materials and Methods

### Data description, analysis and preprocessing

#### Data description and analysis

The patient dataset used in this study was provided by NACC. The data includes patients’ demographics, health history, physical information, elements of the CDR scale^34,35^, Geriatric Depression Scale (GDS)^36–40^, and Functional Activities Questionnaire (FAQ)^41–43^. The detailed information about these feature categories is provided in the Supplementary Table S1.

**Figure 1:**
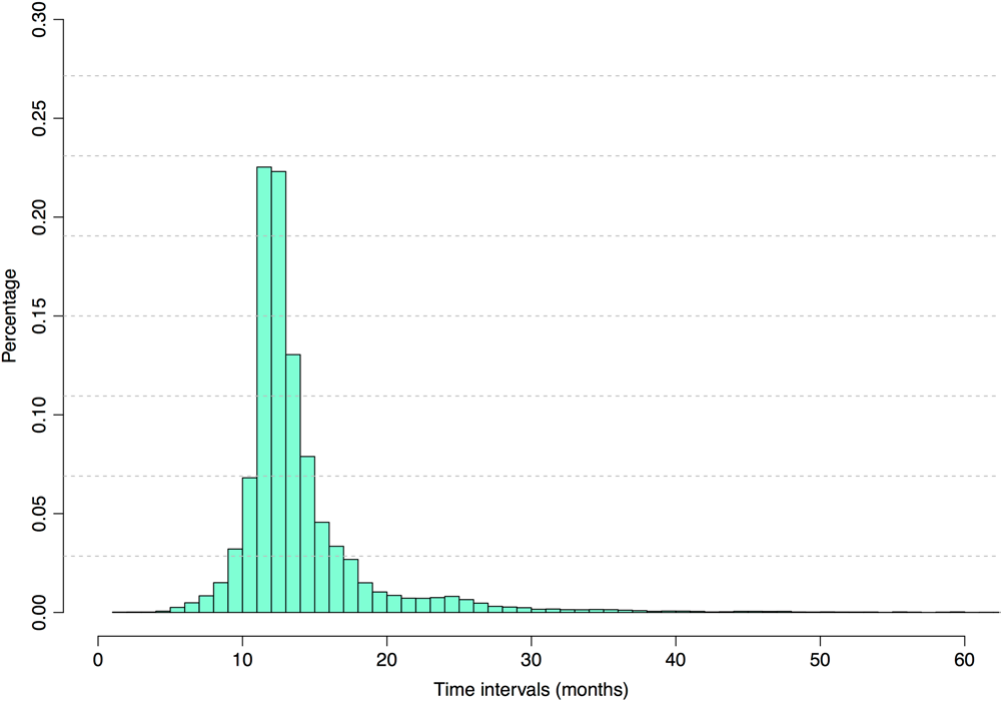
The distribution of time intervals of two successive medical visits.

To focus on the future progression predictive modeling of AD, patients who were marked with probable AD and had more than three visits were chosen from the original dataset. As a result, 5432 patients with probable AD between August 31, 2005 and May 25, 2017 as a subset of the NACC dataset are included in this study. The number of visits still varies with patients in the subset, with an average of 4.98 visits for a patient while the maximal number reaches as high as 12. In addition, the time intervals between patients’ two consecutive visits are quite different too. As illustrated in Fig. 1, although about 46% of time intervals are about 12 months, other time intervals span from 5 months to as far as 5 years.

Table 1 shows the likelihood that a patient changing from one Global CDR score to another between two consecutive visits in the dataset under study. ${{y}}_{{{t}}_{{n}}}$ denotes the global CDR score of a patient in its ${n}^{th}$ visit and ${y}_{{t}_{n+1}}$ denotes the global CDR score of a patient in its ${(n+1)}^{th}$ visit.

With about 60% of visits, the global CDR score of a patient had no change at a given stage, except for the fifth stage (${{y}}_{{{\boldsymbol{t}}}_{{\boldsymbol{n}}}}=3$). With about 40% of visits, a patient got worsen by one stage, and with about 4% of visits, a patient got worsen by two stages. By contrast, with about 4% of visits, a patient got better by one stage with respect to the global CDR score. In short, Table 1 clearly shows that AD progresses slowly over time. Therefore, to help an AD patient with effective healthcare guidance, it is critical to identify if the patient will have pejorative progression in his/her next visit.

**Table 1:** Ratios of Global CDR scores that are changed between two consecutive visits.

Global CDR score	${{\boldsymbol{y}}}_{{{\boldsymbol{t}}}_{{\boldsymbol{n}}{\boldsymbol{+}}1}}={\bf{0}}$	${{\boldsymbol{y}}}_{{{\boldsymbol{t}}}_{{\boldsymbol{n}}{\boldsymbol{+}}1}}={\bf{0.5}}$	${{\boldsymbol{y}}}_{{{\boldsymbol{t}}}_{{\boldsymbol{n}}{\boldsymbol{+}}1}}={\bf{1}}$	${{\boldsymbol{y}}}_{{{\boldsymbol{t}}}_{{\boldsymbol{n}}{\boldsymbol{+}}1}}={\bf{2}}$	${{\boldsymbol{y}}}_{{{\boldsymbol{t}}}_{{\boldsymbol{n}}{\boldsymbol{+}}1}}={\bf{3}}$
${y}_{{t}_{n}}=0$	0.5286	0.4320	0.0384	0.0010	0
${y}_{{t}_{n}}=0.5$	0.0201	0.6304	0.3151	0.0299	0.0045
${y}_{{t}_{n}}=1$	0.0004	0.0488	0.6134	0.2982	0.0392
${y}_{{t}_{n}}=2$	0	0.0023	0.0454	0.6165	0.3358
${y}_{{t}_{n}}=3$	0	0	0.0019	0.0254	0.9727

**Figure 2:**
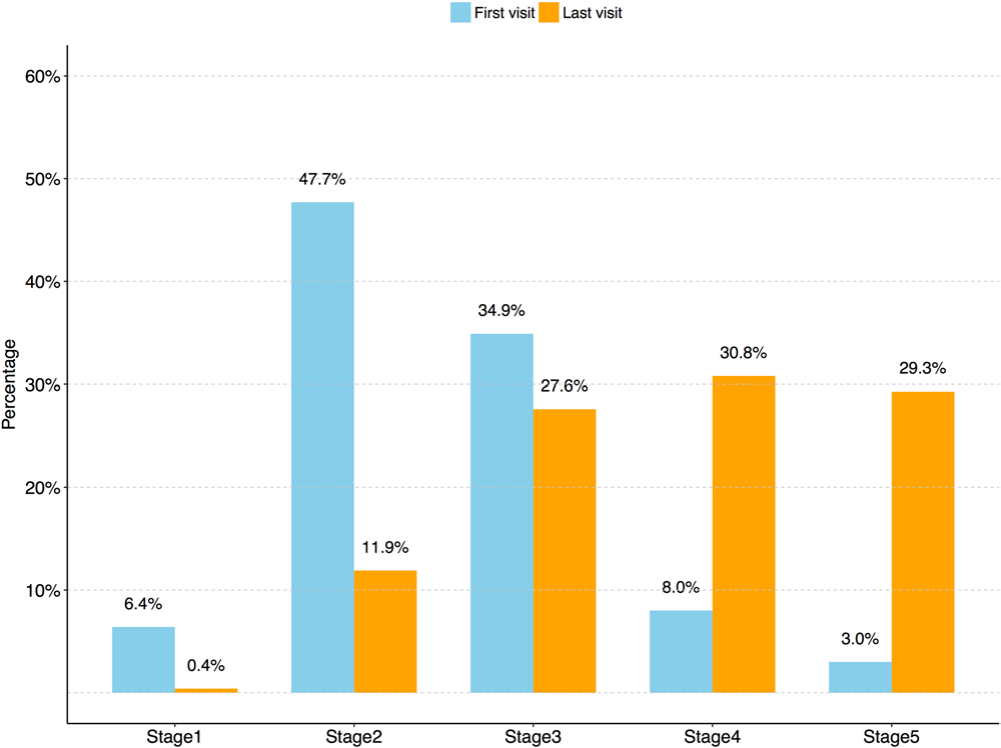
The distribution of patients at different stages with respect to their first visits and last visits.

Figure 2 shows the distribution of demented patients at different progression stages with respect to their first visits and their last visits in the selected subset. 47.7% and 34.9% patients as the majority are at the second and third stages respectively on their first visits, which indicates that most patients began to visit AD related healthcare service centers when they were at either questionable impairment or mild impairment stages. However, 30.8% and 29.3% patients had progressed to or remained at the fourth and fifth stages respectively on their last visits. In fact, when we simply look at Fig. 2 and compare bars of their first and last visits, those AD patients under study had become more severe as time passed. Therefore, it is important and promising to explore how and predict the disease of an AD patient progresses over time.

In total, there are 78 features/predictors (including *time interval*) and a label (Global CDR score) in the created data subset. The variable *time interval* is derived from two successive visit dates, which is measured in months. Detailed descriptions of other variables are listed in the Supplementary Table S1. There are different data types in this data subset, such as continuous variables, ordinal variables, and nominal variables (including dummy variables). Most variables have different percentages of missing data, and the distributions of those missing data are given in Table 2. Thus, it needs to perform data imputations to obtain quality data for predictive modeling.

**Table 2:** Missing data analysis.

Ratio of missing data	Number of Variables
< = 5%	29
5% < and < = 10%	21
10% < and < = 15%	4
15% < and < = 20%	6
20% < and < = 25%	6
25% < and < = 30%	13

#### Data preprocessing

Two steps of data preprocessing were adopted: (a) we first take advantage of some simple and fast imputation techniques to impute the missing data to obtain high quality data, (b) we then apply normalization or encoding on the imputed data to get appropriate inputs for the adopted deep learning model.

In general, we can apply mean imputation to continuous variables, median imputation to ordinal variables, and mode imputation to nominal variables. Nevertheless, in this study we also applied customized imputation schemes to certain variables by taking their feature characteristics into consideration as shown in Table 3.

**Table 3:** Imputation schemes for missing data.

Characteristics of variables	Continuous variables	Ordinal variables	Nominal variables
The variable would not change with different visits for a patient	Imputed with the mean value of first visits of all the patients	Imputed with the median value of first visits of all the patients	Imputed with the mode value of first visits of all the patients
The variable would change with different visits for a patient	Imputed with the mean value of all the visits of all the patients	Imputed with the median value of all the visits of all the patients	Imputed with the mode value of all the visits of all the patients
The variable would change with different visits for a patient and is only related to a specific patient	Imputed with the mean value of other visits of the same patient	Imputed with the median value of other visits of the same patient	Imputed with the mode value of other visits of the same patient

Then normalization or encoding was performed on the imputed data. For the continuous variables, we normalized the variables with their corresponding mean and variance. For the categorical variables (including ordinal and nominal variables), one-hot encoding was performed on each of those variables. For example, if a categorical variable had five classes, we used five-dimension one-hot vectors to represent the variable. After the completion of one-hot encoding for the categorical variables, the dimension of the input features for the proposed model was increased to 234.

### RNN model for AD progression stage prediction

#### Long short-term memory (LSTM) RNN model

**Figure 3:**
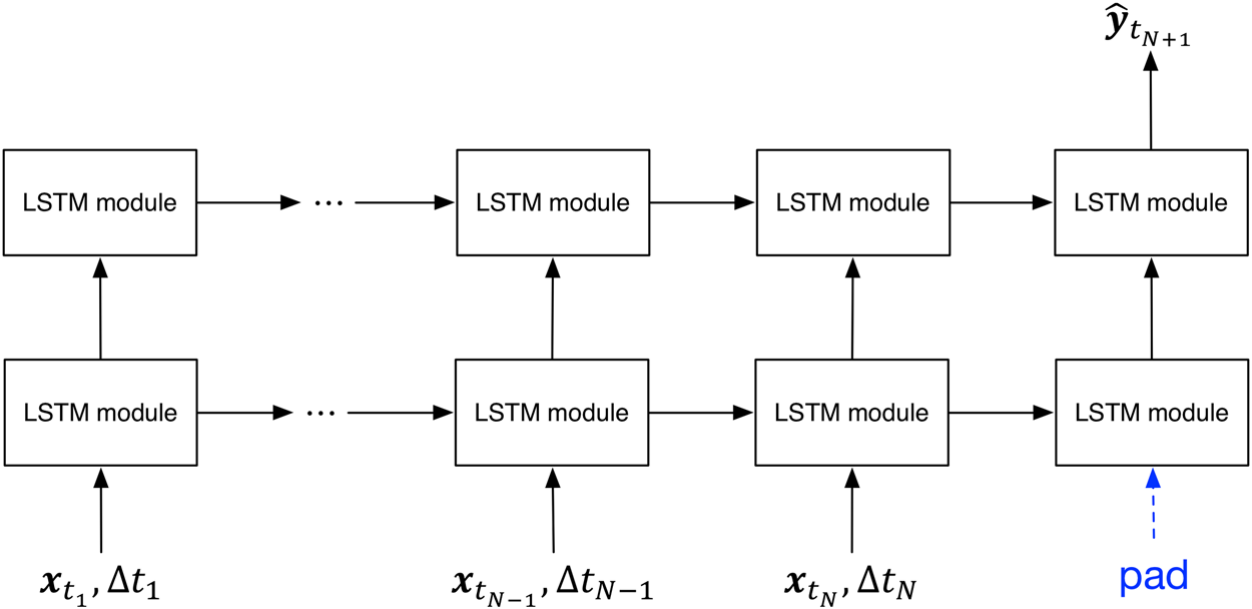
The architecture of the proposed RNN model for AD stage prediction.

We applied RNN to the AD progression modeling in this study. Different from traditional neural networks, RNN models allow temporal information to be passed from one time step to the next time step in the network^23^. The proposed RNN model is structured with one input layer, two hidden layers (Fig. 3), and one output layer. The input layer can accommodate well the needed information of all historical visits and irregular visit time intervals for patients.

Formally, given that there are *N* visits (time points or steps) for a patient, the input at time ${t}_{n}$, $n=1,\,2,\cdots ,\,N$, includes patient’s features ${{\boldsymbol{x}}}_{{t}_{n}}$ and time interval ${\rm{\Delta }}{t}_{n}={t}_{n+1}-{t}_{n}$. More specifically, ${{\boldsymbol{x}}}_{{t}_{n}}$ is a row vector with $Q\,$dimensions where *Q* indicates the number of input features. Note that a feature can be continuous, ordinal or nominal. If the *N*^th^ visit represents the current visit of a patient, then the $(N+1){}^{{\rm{th}}}$ visit represents the next visit of the patient. The prediction model proposed in our study can be written as:

1 $${\hat{y}}_{{t}_{N+1}}=f({x}_{{t}_{1}},{x}_{{t}_{2}},\ldots ,{x}_{{t}_{N}};{\rm{\Delta }}{t}_{1},{\rm{\Delta }}{t}_{2},\cdots ,{\rm{\Delta }}{t}_{N})$$

where the output ${\hat{{\boldsymbol{y}}}}_{{t}_{N+1}}$ is the AD stage of the next visit of the patient (Fig. 3) and the function *f*(·) represents the proposed model. When the prediction model is applied in the real world, ${\rm{\Delta }}{t}_{N}$ can be set according to a user’s need, which means the future prediction time point can be customized as needed. As mentioned earlier, demented patients will experience five stages with reference to CDRGLOB. Therefore, ${\hat{{\boldsymbol{y}}}}_{{t}_{N+1}}$ is a one-hot vector with five dimensions, and each dimension indicates a corresponding AD stage. Since the proposed model has a time window shift between the input and the output, a pad with zero vectors for the (*N* + 1)^th^ visit was performed on the input to create a “many-to-one” model^24^.

**Figure 4:**
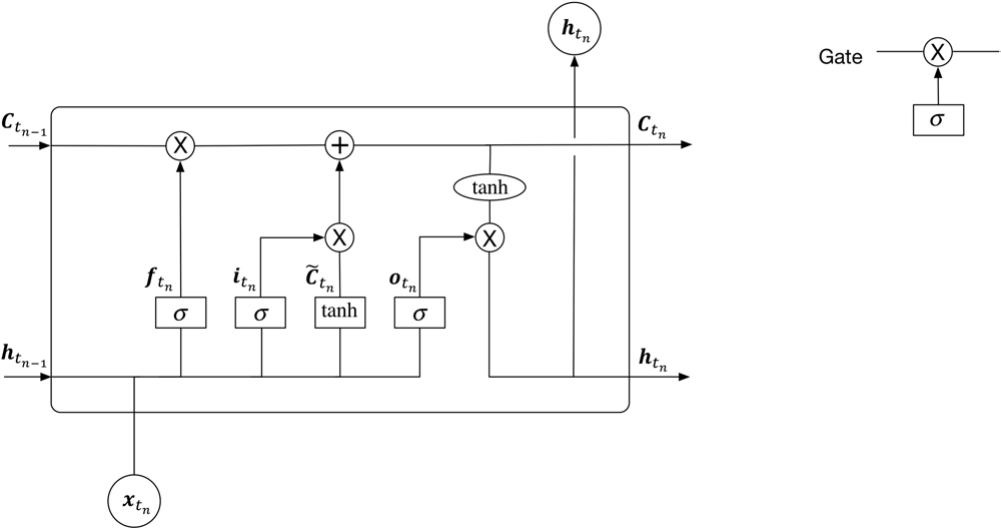
A LSTM module in the proposed RNN model.

Each module in the proposed RNN architecture is a very special kind of RNN cell, called LSTM (Fig. 4)^44,45^. ${{\boldsymbol{C}}}_{{t}_{n-1}}$ is the cell state of the last time step of the same hidden layer, and ${{\boldsymbol{h}}}_{{t}_{n-1}}$ is the output of the last cell state of the same hidden layer. ${{\boldsymbol{C}}}_{{t}_{n}}$ is the cell state of the current time step, and will connect to the next time step of the same hidden layer. ${{\boldsymbol{h}}}_{{t}_{n}}$ is the output part of the current time step, which will pass information to both the next time step of the same hidden layer and the same time step of the next hidden layer, as shown in Fig. 3.

The equations of Fig. 4 are defined as follows^23,24,45^:

2 $${{\boldsymbol{f}}}_{{t}_{n}}=\sigma ({{\boldsymbol{W}}}_{f}\cdot [{{\boldsymbol{h}}}_{{t}_{n-1}},{{\boldsymbol{x}}}_{{t}_{n}}]+{{\boldsymbol{b}}}_{f})$$

3 $${{\boldsymbol{i}}}_{{t}_{n}}=\sigma ({{\boldsymbol{W}}}_{i}\cdot [{{\boldsymbol{h}}}_{{t}_{n-1}},{{\boldsymbol{x}}}_{{t}_{n}}]+{{\boldsymbol{b}}}_{i})$$

4 $${\tilde{{\boldsymbol{C}}}}_{{t}_{n}}=\,\tanh ({{\boldsymbol{W}}}_{C}\cdot [{{\boldsymbol{h}}}_{{t}_{n-1}},{{\boldsymbol{x}}}_{{t}_{n}}]+{{\boldsymbol{b}}}_{C})$$

5 $${{\boldsymbol{C}}}_{{t}_{n}}={{\boldsymbol{f}}}_{{t}_{n}}\,\ast \,{{\boldsymbol{C}}}_{{t}_{n-1}}+{{\boldsymbol{i}}}_{{t}_{n}}\,\ast \,{\tilde{{\boldsymbol{C}}}}_{{t}_{n}}$$

6 $${{\boldsymbol{o}}}_{{t}_{n}}=\sigma ({{\boldsymbol{W}}}_{o}\cdot [{{\boldsymbol{h}}}_{{t}_{n-1}},{{\boldsymbol{x}}}_{{t}_{n}}]+{{\boldsymbol{b}}}_{o})$$

7 $${{\boldsymbol{h}}}_{{t}_{n}}={{\boldsymbol{o}}}_{{t}_{n}}\,\ast \,\tanh ({{\boldsymbol{C}}}_{{t}_{n}})$$

There are three gates in an LSTM cell (Fig. 4): ${{\boldsymbol{f}}}_{{t}_{n}}$, ${{\boldsymbol{i}}}_{{t}_{n}}$ and ${{\boldsymbol{o}}}_{{t}_{n}}$ represents a forget gate, an input gate and an output gate, respectively. ${\tilde{{\boldsymbol{C}}}}_{{t}_{n}}$ represents the candidate value of a new cell. The new cell state, ${{\boldsymbol{C}}}_{{t}_{n}}$, is derived from the cell state of the last time step through the forget gate while the candidate value derived from the input gate. The output part of the current cell state, i.e., ${{\boldsymbol{h}}}_{{t}_{n}}$, is obtained from a tanh (·) activation function.

The proposed RNN model has a chain of the same LSTM module in each hidden layer. Hence, to predict the AD stage of the $(N+1){}^{{\rm{th}}}$ visit for a patient, we used a softmax function:

8 $${\hat{{\boldsymbol{y}}}}_{{t}_{N+1}}=\mathrm{softmax}\,({\bf{W}}\cdot {{\boldsymbol{h}}}_{{t}_{N}}^{(2)}+{\bf{b}})$$

where ${{\boldsymbol{h}}}_{{t}_{N}}^{(2)}$ represents the output of the *N*^th^ step of the second hidden layer.

#### Loss function with regularization

To avoid overfitting, regularization was applied. Formally, the loss function of the model becomes ${\rm{J}}({\boldsymbol{\theta }})+{\rm{\lambda }}\cdot {\rm{\Omega }}(\,\cdot \,)$, where ${\boldsymbol{\theta }}$ indicates the vector of parameters of the model (including weights and biases) and ${\rm{J}}({\boldsymbol{\theta }})$ is the original loss function (before regularization). The regularization item, Ω(·), represents the model complexity while λ is the ratio of the cost of the model complexity and the total cost. In general, the complexity of a model depends on weights. So the regularization item can be simply computed based on weights, i.e., ${\rm{\Omega }}({\bf{W}})$. There are two common kinds of regularization: L1 regularization, and L2 regularization^46^.

9 $${\rm{L}}1:{\rm{\Omega }}({\bf{W}})=\sum _{i}|{w}_{i}|$$

10 $${\rm{L}}2:{\rm{\Omega }}({\bf{W}})=\sum _{i}{w}_{i}^{2}$$

Both L1 and L2 regularization techniques focus on limiting the weight values to avoid overfitting. L1 regularization will make some weights zeroes in the training process, which is equivalently considered as conducting feature selections. While L2 regularization will not make some weights zeroes in the training process. When some weights have small values, their corresponding square values will become smaller, which is small enough to ignore. In this study, L2 regularization is used so that all features can be well considered. The cross-entropy function with regularization for M patients thus is defined as follows:

11 $$L({\boldsymbol{W}},b)=\,-\,\sum _{m=1}^{M}({{\boldsymbol{y}}}_{{t}_{N+1}}^{(m)}log\,{\hat{{\bf{y}}}}_{{t}_{N+1}}^{(m)}+(1-{{\boldsymbol{y}}}_{{t}_{N+1}}^{(m)})log(1-{\hat{{\bf{y}}}}_{{t}_{N+1}}^{(m)}))\,+\,\lambda \cdot \sum _{i}{w}_{i}^{2}$$

where *M* is the total number of patients, ${{\boldsymbol{y}}}_{{t}_{N+1}}^{(m)}$ indicates the true AD stage of the *m*^th^ patient, ${\hat{{\bf{y}}}}_{{t}_{N+1}}^{(m)}$ is the value predicted by the model, λ is a hyperparameter that controls the L2 regularization, and ${w}_{i}$ denotes a weight in the built network.

#### Model evaluation

Our goal is to predict the AD progress of a patient at the time he/she visits in the future. Hence, accuracy that defines the percentage of correct predictions can be used as the basic metric to evaluate the performance of a multi-class prediction model. To a patient, he/she might be more concerned with the correct prediction of his/her AD progression with respect to CDRGLOB. In practice, an accurate prediction of disease progression provides a significant value to the patient, doctors, and caregivers.

Based on the definition of CDRGLOB, if the value of a patient’s CDRGLOB from the *N*^th^ visit to the (*N* + 1)^th^ visit increases by 0.5, 1, 2 or 3, we consider that the patient’s AD has developed or made pejorative progress. Otherwise, we simply consider that the patient’s condition has been stable or not deteriorated. In theory, if the value of CDRGLOB of the patient changes by any negative number, the patient would have got better. We know this wouldn’t happen until an effective drug or treatment is discovered. Thus, the pejorative progression identification accuracy (PPIA) is a meaningful and important metric for now.

In addition, patients can be divided into two different groups with respect to CDRGLOB. One group consists of the patients whose CDRGLOB values are 0, 0.5, or 1 on their last visits; the other group includes the patients whose CDRGLOB values are 2 or 3 on their last visits. Obviously, the second group includes severe AD patients, while the first group is considered as a non-severe one. Thus, the prediction accuracy of the second group can be denoted as severe patient identification accuracy (SPIA), which is another performance metric for the proposed model that users may also have interest.

### Model training and comparisons

#### Baseline models for performance comparisons

For the progression prediction problem under study, one may wonder if classical time series methods might be applied to solve the problem. That is, one might use the information of the target variable (i.e., AD stage) of historical visits to predict its future values, i.e., ${\hat{{\boldsymbol{y}}}}_{{t}_{N+1}}=\psi ({{\boldsymbol{y}}}_{{t}_{1}},{{\boldsymbol{y}}}_{{t}_{2}},\,\ldots ,{{\boldsymbol{y}}}_{{t}_{N}})$, where $\psi (\,\cdot \,)$ represents a classical time series method. Unfortunately, as mentioned earlier, the number of visits has an average of 4.98 visits for a patient and the target variable is an ordinal variable. As a result, traditional time series methods fail to capture the AD progression patterns. In other words, traditional time series methods can’t be well applied to the problem under study. Therefore, four machine-learning algorithms were chosen as baselines in this study. These four baseline models include logistic regression (LR), support vector machine (SVM), decision tree (DT), and random forest (RF), which will be used to be compared with the proposed RNN model in terms of their performances. Since these baseline models cannot handle various lengths of longitudinal temporal data, three training approaches are adopted for each of the baseline models to predict AD patients’ future progression stages.

Approach 1: we can train the baseline models with aggregated features of all patients’ historical visits, which is a common method for longitudinal data in predictive modeling^27^. The aggregated features’ values from all the historical visits can be defined as:

12 $${\bar{{\boldsymbol{x}}}}_{{t}_{1},\cdots ,{t}_{N}}=({\bar{{\boldsymbol{x}}}}_{1},\,{\bar{{\boldsymbol{x}}}}_{2},\cdots ,{\bar{{\boldsymbol{x}}}}_{Q})$$

where ${\bar{{\boldsymbol{x}}}}_{{t}_{1},\cdots ,{t}_{N}}\,$is a vector with *Q* dimensions, and the aggregated value of the *q*^th^ dimension ($q=1,\,2,\,\cdots ,Q$) is defined as follows:

- If the *q*^th^ feature is a continuous type variable, then the aggregated value ${\bar{{\boldsymbol{x}}}}_{q}$ is the mean of the feature values of all the historical visits.
- If the *q*^th^ feature is an ordinal type variable, then the aggregated value ${\bar{{\boldsymbol{x}}}}_{q}$ is the median of the feature values of all the historical visits.
- If the *q*^th^ feature is a nominal type variable, then the aggregated value ${\bar{{\boldsymbol{x}}}}_{q}$ is the mode value of the feature values of all the historical visits.

Based on the aggregated features’ values, the baseline models used to predict the future progression stage can be defined as follows:

13 $${\hat{{\boldsymbol{y}}}}_{{t}_{N+1}}={f}_{1}({\bar{{\boldsymbol{x}}}}_{{t}_{1},\cdots ,{t}_{N}})$$

where ${f}_{1}(\,\cdot \,)$ represents a baseline model.

Approach 2: similar to time series methods, the baseline models can also be implemented with the information of the most recent *r* visits among historical visits. We merge the feature values of most recent *r* visits into one feature vector, i.e.,

14 $${\tilde{{\boldsymbol{x}}}}_{{t}_{N-r+1},\cdots ,{t}_{N}}=\,({{\boldsymbol{x}}}_{{t}_{N-r+1}},{{\boldsymbol{x}}}_{{t}_{N-r+2}},\,\ldots ,{{\boldsymbol{x}}}_{{t}_{N}})$$

where ${\tilde{{\boldsymbol{x}}}}_{{t}_{N-r+1},\cdots ,{t}_{N}}$ is a merged vector with r*Q dimensions, note that ${{\boldsymbol{x}}}_{{t}_{N-r+1}}$, ${{\boldsymbol{x}}}_{{t}_{N-r+2}},\,\,\ldots ,\,{{\boldsymbol{x}}}_{{t}_{N}}$ are feature vectors with *Q* dimensions at the $(N-r+1){}^{{\rm{th}}}$, the $(N-r+2){}^{{\rm{th}}}$, $\ldots$, and the *N*^th^ visit, respectively.

Based on the merged feature vector, the baseline models used to predict the future progression stage can be then defined as follows:

15 $${\hat{{\boldsymbol{y}}}}_{{t}_{N+1}}={f}_{2}({\tilde{{\boldsymbol{x}}}}_{{t}_{N-r+1},\cdots ,{t}_{N}})$$

where ${f}_{2}(\,\cdot \,)$ represents a baseline model and *r* should be smaller than the minimum number of visits of patients. Note that the minimal number of visits among patients is 3 in the dataset under study, and in this study *r* is therefore set to 2 in order to evaluate the prediction performance of built models. That is, one visit was left for each patient that was used to evaluate the prediction performance of the models.

Approach 3: the baseline models can be easily implemented with the feature information at the *N*^th^ visit, i.e.,

16 $${\hat{{\boldsymbol{y}}}}_{{t}_{N+1}}={f}_{3}({{\boldsymbol{x}}}_{{t}_{N}})$$

where ${f}_{3}(\,\cdot \,)$ represents a baseline model.

Regarding the above training approaches, Approach 3 is essentially a special case of Approach 2. As mentioned above, r is set to 2 for Approach 2 in this study, which makes Approach 2 and Approach 3 very similar. It is worth mentioning that when the minimum number of visits of patients increases, r can be set to other higher values.

Different from the baseline models, we can train our proposed model with or without time intervals (TI) that are derived from two consecutive visits. Essentially, we aim to investigate whether the proposed RNN model can fully leverage irregular time intervals information to improve AD progression modeling performances.

#### Impact analysis of various feature categories

To get a better understanding of factors or variables impacting the cognitive declines of patients, we perform feature analysis based on different subsets of variables. More specifically, more experiments are conducted to investigate how each category of features would impact the prediction performance. In this study, the full dataset based model (or simply called the full model) contains 6 feature categories: Subject Demographics, Subject Health History, Physical information, CDR, GDS, and FAQ. Since the visit time intervals, demographical, health historical physical information and MMSE score are basic information for patients, we conduct various sub-models using different combinations of CDR, GDS and/or FAQ to analyze their impacts on corresponding modeling performances.

### Data availability

The data that support the findings of this study are available from NACC but restrictions apply to the availability of these data, which were used under license for the current study, and so are not publicly available. Data are however available from the authors upon reasonable request and with permission of NACC.

## Results

In this study, we implemented our RNN model of 2 hidden layers with 100 hidden units at each layer. The learning rate decay and moving average decay mechanism were applied in the training process. L2 regularization was added to the loss function, and Adam Optimizer was used in the loss function optimization^47^. We used 10-fold cross validation in our experiments. Thus the results illustrated later are the average values of the 10-fold cross validation runs. The detailed implementation information for the proposed model is provided in the Supplementary File.

First, we conducted different experiments to train the proposed model with or without TIs. Then we compared the performances of the proposed models with the baseline models. The results are shown in Table 4.

**Table 4:** The performance comparison of the proposed models and the baseline models.

Models	Accuracy	PPIA	SPIA
LSTM with TI	**0.9906 **±** 0.0043**	**0.9894 **±** 0.0074**	**0.9912 **±** 0.0039**
LSTM w/o TI	0.9843 ± 0.0057	0.9792 ± 0.0117	0.9849 ± 0.0053
LR with average aggregation	**0.7955 **±** 0.0216**	**0.7126 **±** 0.0345**	**0.7986 **±** 0.0211**
LR with two most recent visits	0.6652 ± 0.0162	0.5057 ± 0.0409	0.6674 ± 0.0163
LR with the most recent visit	0.6803 ± 0.0243	0.5209 ± 0.0370	0.6825 ± 0.0243
SVM with average aggregation	**0.7445 **±** 0.0237**	**0.6465 **±** 0.0516**	**0.7468 **±** 0.0226**
SVM with two most recent visits	0.6533 ± 0.0165	0.4825 ± 0.0445	0.6552 ± 0.0163
SVM with the most recent visit	0.6746 ± 0.0209	0.4931 ± 0.0245	0.6757 ± 0.0208
DT with average aggregation	**0.7035 **±** 0.0206**	**0.6223 **±** 0.0267**	**0.7058 **±** 0.0200**
DT with two most recent visits	0.5810 ± 0.0199	0.4463 ± 0.0470	0.5829 ± 0.0196
DT with the most recent visit	0.5916 ± 0.0204	0.4705 ± 0.0458	0.5934 ± 0.0196
RF with average aggregation	**0.6916 **±** 0.0223**	**0.5786 **±** 0.0487**	**0.6944 **±** 0.0227**
RF with two most recent visits	0.6373 ± 0.0181	0.4517 ± 0.0430	0.6399 ± 0.0179
RF with the most recent visit	0.6416 ± 0.0183	0.4570 ± 0.0422	0.6441 ± 0.0186

LSTM with TI and LSTM w/o TI are implemented based on the dataset of patients with more than 3 visits. Baseline models with average aggregation are trained with aggregated features derived from the longitudinal data. Baseline models with two most recent visits are trained directly with the information of the $({N}-1){}^{{\rm{th}}}$ visit and the ***N***^th^ visit among historical visits. Baseline models with the most visit are trained directly with the *N*^th^ visit. *LSTM with TI* model and all the baseline models are trained with time intervals, while *LSTM w/o TI* is trained without time intervals. Note that the results presented here are mean values and the standard deviation values of the 10-fold cross validation, and the performances of each fold are provided in the Supplementary Tables S2–S7.

As shown in Table 4, *LSTM with TI* performs better than *LSTM w/o TI* by about 0.6%, 1.0% and 0.6% regarding the metrics - Accuracy, PPIA, and SPIA, respectively. In other words, a model of AD progression with irregular TI is better than one only with logical temporal information. This confirms that LSTM based RNN models can truly account for the longitudinal temporal patterns in the problem under study.

Note that the baseline models that are trained with the aggregated information of all the historical visits outperform the corresponding models based on these methods trained with the information of two most recent historical visits or the most recent historical visit. The proposed models based on LSTM (both *LSTM with TI* and *LSTM w/o TI*) are far superior to the baseline models as shown in Table 4. Specially, the accuracy of *LSTM with TI* is about 19.5%, 24.6%, 28.7% and 29.9% better than LR, SVM, DT and RF models trained with the aggregated information of all the historical visits, respectively. Moreover, the proposed models also perform far better than the baseline models with respect to other two metrics PPIA and SPIA.

In addition, to analyze the impact of various feature categories on the model performance, we conducted experiments of *LSTM with TI* using different combinations of feature categories (Table 5).

**Table 5:** Results of the *LSTM with TI* model trained with different combinations of feature categories.

Models	Accuracy	PPIA	SPIA
Full Model	**0.9906 **±** 0.0043**	**0.9894 **±** 0.0074**	**0.9912 **±** 0.0039**
Model without CDR	0.9557 ± 0.0121	0.9470 ± 0.0273	0.9565 ± 0.0125
Model without GDS	0.9900 ± 0.0057	0.9832 ± 0.0103	0.9906 ± 0.0054
Model without FAQ	0.9615 ± 0.0140	0.9429 ± 0.0262	0.9624 ± 0.0143
Model without CDR, GDS	0.9699 ± 0.0094	0.9637 ± 0.0154	0.9708 ± 0.0091
Model without CDR, FAQ	0.7191 ± 0.0282	0.6867 ± 0.0471	0.7207 ± 0.0278
Model without GDS, FAQ	0.9689 ± 0.0144	0.9620 ± 0.0229	0.9698 ± 0.0141
Model without CDR, GDS, FAQ	0.7148 ± 0.0355	0.6868 ± 0.0437	0.7147 ± 0.0353

Note that the results presented here are mean values and the standard deviation values of the metrics for the 10-fold cross validation, and the performances of each fold are provided in the Supplementary Tables S8–S14.

As illustrated in Table 5, a model without GDS shows a slight negative impact on the model’s capability to predict, while a model without CDR or FAQ performs much worse than the full model, with accuracy being decreased by about 3.5% or 2.9% respectively. Note that the performance is considerably decreased when CDR and FAQ are not considered by the model at the same time. Therefore, when the model is applied in practice, it is very important to take into account CDR data and/or FAQ data, and if available, both CDR and FAQ should be included for better results.

To further show the effect of each specific feature in the CDR/FAQ category on the model performance, experiments with variable control approaches have been conducted. A model only with the basic information (i.e., a model without CDR, FAQ, GDS) is defined as our basic model. There are 9 features in the CDR category and 10 features in the FAQ category. Each time one specific feature of the CDR/FAQ category was incorporated into the basic model to investigate the impact of the specific feature on the model performance. Table 6 shows the model improvement by incorporating a specific feature of the CDR/FAQ category when compared with the basic model.

**Table 6:** Improvement by incorporating a specific feature of CDR/FAQ category compared with the basic model.

Category	Incorporated Feature	ΔAccuracy	ΔPPIA	ΔSPIA
CDR	HOMEHOBB	**0.1145**	**0.1269**	**0.1124**
CDR	COMMUN	**0.1110**	**0.1201**	**0.1119**
CDR	ORIENT	**0.1106**	**0.1192**	**0.1106**
CDR	JUDGMENT	**0.1098**	**0.1135**	**0.1097**
CDR	CDRSUM	**0.1040**	**0.1101**	**0.1026**
CDR	MEMORY	0.0905	0.1083	0.0920
FAQ	GAMES	0.0844	0.1017	0.0825
CDR	PERSCARE	0.0817	0.1001	0.0820
FAQ	BILLS	0.0776	0.0990	0.0756
FAQ	REMDATES	0.0764	0.0929	0.0728
FAQ	MEALPREP	0.0731	0.0918	0.0723
FAQ	TAXES	0.0729	0.0914	0.0717
FAQ	STOVE	0.0717	0.0881	0.0679
FAQ	TRAVEL	0.0686	0.0825	0.0647
FAQ	SHOPPING	0.0631	0.0773	0.0621
FAQ	PAYATTN	0.0497	0.0650	0.0548
CDR	COMPORT	0.0350	0.0476	0.0329
FAQ	EVENTS	0.0311	0.0472	0.0321
CDR	CDRLANG	0.0225	0.0366	0.0147

Note that the results presented here are the mean differences from their 10-fold cross-validation runs. The mean values and the standard deviations for the 10-fold cross-validation runs are provided in the slementary Table S15.

As shown in Table 6, the values of all the three metrics that had more than 10% improvements are marked in bold. It is clear that HOMEHOBB, COMMUN, ORIENT, JUDGMENT, and CDRSUM in the CDR category significantly contribute to the model performance improvement. Note that the feature CDRSUM is actually the sum of the scores of six features including MEMORY, ORIENT, JUDGMENT, COMMUN, HOMEHOBB, PERSCARE. Therefore, HOMEHOBB, COMMUN, ORIENT, and JUDGMENT as independent features are ones that substantially impact the model performance. By contrast, top contributed features in the FAQ category help improve the model performance by about 7% respectively, which are less significant when compared to top contributed CDR features.

## Discussion

As indicated in Table 4 the proposed LSTM RNN model when it is trained with irregular TIs performs better than one without accounting for TIs. Generally, the proposed model performs far better than models based on traditional machine learning methods such as LR, SVM, DT and RF. This mainly attributes to the fact that the proposed model can fully capture and leverage patients’ temporal information patterns along with their historical visits.

Interestingly, the results of the feature analysis show that CDR and FAQ play an important role in the AD progression prediction, while GDS contains less effective predictors when compared to CDR and FAQ. In fact, these findings are in line with the medical implications of these data categories:

- CDR includes six cognitive elements in the standard CDR scale version and two additional variables defining the language and the behavior-comportment and personality of a patient. Together they can comprehensively describe the clinical dementia status of patients^35^.
- FAQ is used to access a patient’s instrumental activities of daily living (IADLs), which is effective when used to monitor the functional change of the patient over time^41–43^. For an individual with AD, his/her functional ability usually declines over time. Hence, FAQ has been well considered as risk prognosis for cognitive impairment^48–50^.
- GDS is a self-administered instrument, so when a person with mild dementia of the Alzheimer’s type, GDS is not an effective tool to identify depression^37^. Specially, when a patient is not able to complete a GDS test according to clinician’s best judgment, the corresponding GDS items will be marked as “did not answer”, which essentially become missing data in the archived database. Hence, GDS variables are not considered as effective predictors in the AD progression modeling.

The feature analysis mentioned earlier in fact provides sufficient evidences that are aligned well with the intentions or implications of these data categories.

To the best of our knowledge, we are the first group to apply RNN to the AD progression predictive modeling. The contributions of this study can be summarized as follows:

- First, unlike classification modeling, without a patient’s next visit feature information the proposed LSTM RNN model can predict his/her next visit’s AD progression stage. This is attributed to the adopted RNN with an enhanced structure. Because we have successfully incorporated the shift of time steps into the proposed model, the longitudinal medical patterns of his/her previous visits on record can be well captured and leveraged.
- Secondly, because the “many-to-one” RNN structure is applied, the prediction can also be executed without target variables (i.e., AD stages in this study) in the time steps that are marked as preceding visits from the very last one. In other words, only the values of the target variable in the last time step are needed in a developed model based on the adopted RNN structure. Therefore, the adopted RNN structure for this kind of study works well for the target variables that are partially or totally missing for the preceding time steps except the very last ones of patients in the dataset.
- Thirdly, the proposed progression prediction model can be very adaptive over time since it allows irregular visit TIs and various numbers of visits. Note that TIs are always irregular or uneven, which frequently depends on the patient’s preference or need. It is worth mentioning that the prediction time interval between the current visit and the next visit is part of the input information of a model, which can be chosen by users according to their prediction requirements.
- Finally, other metrics defining AD progression stages can be used to replace the Global CDR score if necessary. The severity stages of AD progressions can be adjusted for a variety of metrics with different granularity levels. Furthermore, the proposed model can be applied to other chronic disease progression modeling.

In the near future, we will develop models that can account for deeper and more granular details in patients’ information, for example different deteriorating rates among patients. If possible, more accurate classification schemes (e.g., biomarker indicators) will be taken into account. Furthermore, we will extend the proposed approach to broader studies with a focus on early disease stage predictions, such as exploring how patients progress from non-demented to demented stages.

## Conclusion

This paper proposed a disease progression model based on a deep RNN with LSTM cells. A “many-to-one” architecture with enhancements in support of time step shifts was fully leveraged in this study. As a result, the proposed model can accurately predict the AD stage of the next visit of a patient when the information of the patient’s previous visits becomes available. By relying on the real-world dataset, i.e., the NACC dataset for patients with AD, we tested and validated the developed model to confirm its applicability in practice, and also compared its performance with four classic approaches. We also explored several sub-models using different combinations of feature categories to analyze their impacts on the model’s performances. In overall, the results of this project show that the proposed LSTM RNN model can effectively predict the future statuses of AD patients. Most promisingly, we can easily apply the proposed model to other chronic disease progression predictive modeling.

## Electronic supplementary material

Supplementary information
