# Supplementary material for: Predictive Modeling of the Progression of Alzheimer’s Disease with Recurrent Neural Networks
Source: Sci Rep. 2018 Jun 15;8:9161. doi: 10.1038/s41598-018-27337-w (PMC6003986; doi:10.1038/s41598-018-27337-w)
Supplement: Supplementary file 1 — Supplementary information [file 41598_2018_27337_MOESM1_ESM.pdf]

Supplementary Information for the Paper:

Predictive Modeling the Progression of Alzheimer's Disease with Recurrent Neural Networks

Tingyan Wang[1, 2], Robin G. Qiu[2*], and Ming Yu[1]

[1] Health Care Services Research Center, Department of Industrial Engineering, Tsinghua University, Beijing 100084, China and [2] Big Data Lab, Division of Engineering and Information Science, The Pennsylvania State University, Malvern, PA 19355, USA

Correspondence and requests for materials should be addressed to R.G.Q. (email: robinqiu@psu.edu)

Detailed data information

Supplementary Table S1 shows the feature categories and their corresponding variables used in this study. Some variables were derived from the original dataset by the authors. Note that the descriptions of most variables are directly extracted from the National Alzheimer's Coordinating Center (NACC) Researcher's Data Dictionary — Uniform Data Set (RDD-UDS). The types of variables were assigned by the authors.

Supplementary Table S1. Feature categories and variable descriptions

**NACC Categories**	**Variable Name**	**Variable Type**	**Descriptions**[1]
**AD stage**	CDRGLOB	Ordinal	Global CDR score, which is used to define the AD progression stages in this study,
**Time interval**	TI	Continuous	Derived by authors;<br>Time interval between two consecutive visits
**A1 Subject Demographics**	AGE	Continuous	Derived by authors
	SEX	Nominal	Subject's sex
	RACE	Nominal	Subject's race
	PRIMLANG	Nominal	Primary language
	EDUC	Ordinal	Years of education
	NACCLIVS	Nominal	Living situation
	INDEPEND	Ordinal	Level of independence
	RESIDENC	Nominal	Type of residence
	MARISTAT	Nominal	Marital status
	HANDED	Nominal	Is the subject left- or right-handed?

[1] National Alzheimer's Coordinating Center (Walter A. Kukull, PhD, Director), Researcher's Data Dictionary — Uniform Data Set (RDD-UDS), version 3.0, March 2015.

NACC Categories	Variable Name	Variable Type	Descriptions[1]
	TOBAC30	Nominal	Smoked cigarettes in last 30 days
A5 Subject Health History	TOBAC100	Nominal	Smoked more than 100 cigarettes in life
	SMOKYRS	Ordinal	Total years smoked cigarettes
	CVHATT	Ordinal	Heart attack/cardiac arrest
	CVAFIB	Ordinal	Atrial fibrillation
	CVANGIO	Ordinal	Angioplasty/endarterectomy/stent
	CVBYPASS	Ordinal	Cardiac bypass procedure
	CVPACE	Ordinal	Pacemaker
	CVCHF	Ordinal	Congestive heart failure
	CVOTHR	Ordinal	Other cardiovascular disease
	CBSTROKE	Ordinal	Stroke
	CBTIA	Ordinal	Transient ischemic attack (TIA)
	SEIZURES	Ordinal	Seizures
	DIABETES	Ordinal	Diabetes
	DIABTYPE		If Recent/active or Remote/inactive Original UDS question v3 diabetes, which type?
	HYPERTEN	Ordinal	Hypertension
	HYPERCHO	Ordinal	Hypercholesterolemia
	B12DEF	Ordinal	Vitamin B12 deficiency
	THYROID	Ordinal	Thyroid disease
	ALCOHOL	Ordinal	Alcohol abuse — clinically significant impairment occurring over a 12-month period manifested in one of the following areas: work, driving, legal, or social Numeric longitudinal
	DEP2YRS	Nominal	Active depression in the last two years
	DEPOTHR	Nominal	Depression episodes more than two years ago
	PSYCDIS	Ordinal	Other psychiatric disorder
	HEIGHT	Continuous	Subject’s height (inches)
B1 Physical	WEIGHT	Continuous	Subject’s weight (lbs)
	BPSYS	Continuous	Subject blood pressure (sitting), systolic
	BPDIAS	Continuous	Subject blood pressure (sitting), diastolic
	HRATE	Continuous	Subject resting heart rate (pulse)
	VISION	Nominal	Without corrective lenses, is the subject’s vision functionally normal?
	HEARING	Nominal	Without a hearing aid(s), is the subject’s hearing functionally normal?
B4 Global Staging Clinical Dementia Rating (CDR)	MEMORY	Ordinal	Memory
	ORIENT	Ordinal	Orientation
	JUDGMENT	Ordinal	Judgment and problem-solving
	COMMUN	Ordinal	Community affairs
	HOMEHOBB	Ordinal	Home and hobbies
	PERSCARE	Ordinal	Personal care

NACC Categories	Variable Name	Variable Type	Descriptions[1]
	CDRSUM	Ordinal	Standard CDR sum of boxes
	CDRLANG	Ordinal	Language
	COMPORT	Ordinal	Behavior, comportment, and personality
	NOGDS	Nominal	Is the subject able to complete the GDS, based on the clinician's best judgment?
**B6 Geriatric Depression Scale (GDS)**	SATIS	Nominal	Are you basically satisfied with your life?
	DROPACT	Nominal	Have you dropped many of your activities and interests?
	EMPTY	Nominal	Do you feel that your life is empty?
	BORED	Nominal	Do you often get bored?
	SPIRITS	Nominal	Are you in good spirits most of the time?
	AFRAID	Nominal	Are you afraid that something bad is going to happen to you?
	HAPPY	Nominal	Do you feel happy most of the time?
	HELPLESS	Nominal	Do you often feel helpless?
	STAYHOME	Nominal	Do you prefer to stay at home, rather than going out and doing new things?
	MEMPROB	Nominal	Do you feel you have more problems with memory than most?
	WONDRFUL	Nominal	Do you think it is wonderful to be alive now?
	WRTHLESS	Nominal	Do you feel pretty worthless the way you are now?
	ENERGY	Nominal	Do you feel full of energy?
	HOPELESS	Nominal	Do you feel that your situation is hopeless?
	BETTER	Nominal	Do you think that most people are better off than you are?
	NACCGDS	Ordinal	Total GDS Score
**B7 Functional Activities Questionnaire (FAQ)**	BILLS	Ordinal	In the past four weeks, did the subject have any difficulty or need help with: Writing checks, paying bills, or balancing a checkbook
	TAXES	Ordinal	In the past four weeks, did the subject have any difficulty or need help with: Assembling tax records, business affairs, or other paper
	SHOPPING	Ordinal	In the past four weeks, did the subject have any difficulty or need help with: Shopping alone for clothes, household necessities, or groceries
	GAMES	Ordinal	In the past four weeks, did the subject have any difficulty or need help with: Playing a game of skill such as bridge or chess, working on a hobby
	STOVE	Ordinal	In the past four weeks, did the subject have any difficulty or need help with: Heating water, making a cup of coffee, turning off the stove
	MEALPREP	Ordinal	In the past four weeks, did the subject have any

NACC Categories	Variable Name	Variable Type	Descriptions[1]
			difficulty or need help with: Preparing a balanced meal
	EVENTS	Ordinal	In the past four weeks, did the subject have any difficulty or need help with: Keeping track of current events
	PAYATTN	Ordinal	In the past four weeks, did the subject have any difficulty or need help with: Paying attention to and understanding a TV program, book, or magazine
	REMDATES	Ordinal	In the past four weeks, did the subject have any difficulty or need help with: Remembering appointments, family occasions, holidays, medications
	TRAVEL	Ordinal	In the past four weeks, did the subject have any difficulty or need help with: Traveling out of the neighborhood, driving, or arranging to take public transportation
	NACCMMSE	Ordinal	Total Mini-Mental State Examination (MMSE) score (using D-L-R-O-W)

Implementation tools

Logistic Regression (LR), Support Vector Machine (SVM), Decision Tree (DT) and Random Forest (RF) models were implemented in the Scikit-learn v0.19.1 with sklearn.linear_model.LogisticRegression, sklearn.svm.LinearSVC, sklearn.tree.DecisionTreeClassifier, and sklearn.ensemble.RandomForestClassifier, respectively. The proposed RNN models were implemented with the Tensorflow.

Implementation information for the proposed RNN model

Input padding

After the data imputation and encoding was completed, the input had 234 dimensions (the dimensions would be 233 for the model without time intervals). The output stayed the same with 5 dimensions. For a patient, the number of his/her visits defines his/her time step dimension. Because the input of each patient to the model requires the same length of

sequences in the time step dimension, zero sequences were padded to the input matrix to get the same length with respect to ones who had the maximum visits. In addition, since the proposed model has a time window shift between the input and the output, a pad with zero vectors for the $(T+1)^{\text{th}}$ visit was performed on the input.

Network parameters initialization

In the constructed network in this study, each hidden unit was an LSTM cell and the Softmax function was used as the activation function of the output layer. The initialization value of each weight of the network was generated from a truncated normal distribution so that a large variance can be avoided. Technically, the initialization value of each weight was first generated from a predefined normal distribution, i.e., $N(\mu, \sigma^2)$. If the generated value was within the range of $[\mu - 2\sigma, \mu + 2\sigma]$, then the value would be taken as the weight initialization value, otherwise the value would be discarded; the initialization process repeated until an appropriate initialization value was obtained. Note that μ and σ were the mean and the standard deviation of the normal distribution, which were set to 0 and 0.001, respectively. The initialization values of biases were constant, which were set to 0.001 in this study.

Hyper parameters for the training process

In this study, learning rate decay and moving average decay mechanisms were used to train the proposed model to achieve the best performance effectively.

When an RNN model is trained, learning rates play a key role in its converging process. Large learning rates will lead to large fluctuation in the values of learned parameters, resulting in that the model has a difficulty in getting converged. While small learning rates will usually result in a slow converging process. Hence, a chosen learning rate should be neither too large nor too small. To tackle the challenge in setting a right learning rate, a flexible learning rate scheme was used in this study. Technically, the training process starts with a base learning rate, and then in each epoch the learning rate decreased exponentially

with a given decay rate. Note that an epoch means that the training process performs completely on the whole training dataset. The training process will keep going until the model gets converged or the training computation reaches the designated maximum of iterations. According to the learning rate decay mechanism that was described above, the base-learning rate was set to 0.01. The learning rate decreased exponentially in the training process with a decay rate 0.96.

Exponential moving average decay mechanism was applied to make the model more robust. In detail, an exponential moving average class was defined for all the training variables, i.e., weights and bias. Moving average decay was applied and accordingly the moving average for the variables got repeatedly updated throughout the training iterations. Note that the decay rate of the moving average mechanism will be responsible for model updating rate. For the initialization, the decay rate of the moving average mechanism is set to 0.99 and it will be adjusted dynamically in the train process. To avoid overfitting, we used L2 regularization by assigning the regularization rate to 0.0001.

Moreover, we used the 10-fold cross-validation to evaluate the predictive model: randomly partition the data into 10 equal size subsets, and 9 subsets are used to train the model and the remaining one is used to test the model, and the process is then repeated 10 times/folds. Then we get the performance of the model by averaging the 10 test results from the folds.

For each fold of the cross validation, we used the mini-batch method for the model training. The batch size was set to 60. We performed 200 epochs on the whole training dataset. In addition, at the beginning of each training epoch, we shuffle training dataset. At the end of each epoch (i.e., a training process has been performed completely on the whole training dataset once and a trained model has been achieved), we use the test dataset to test the trained model to detect the trend of the model performance. The model performance of each fold was derived from the 200th epoch. For example, Figure 1 and Figure 2 show the loss and the accuracy of training data and test data in one specific fold of the 10-fold cross validation for the proposed model *LSTM with TI*.

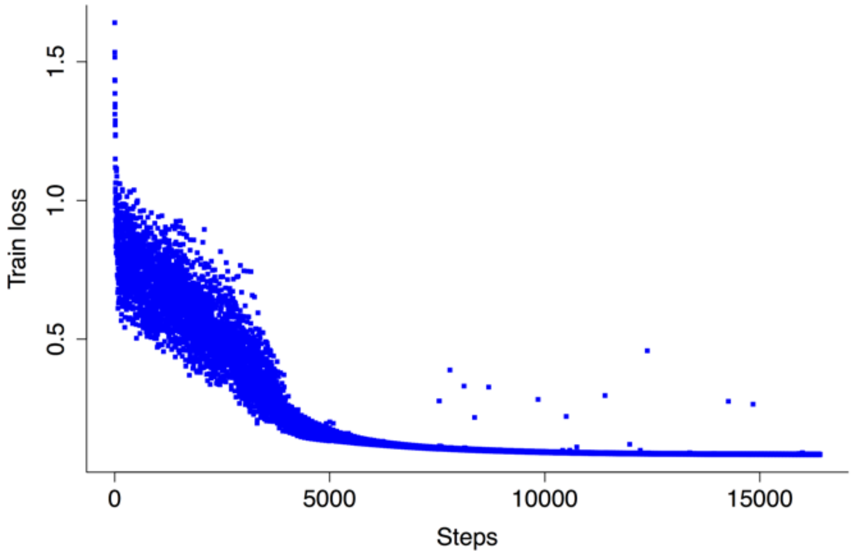

(A) Loss of training data

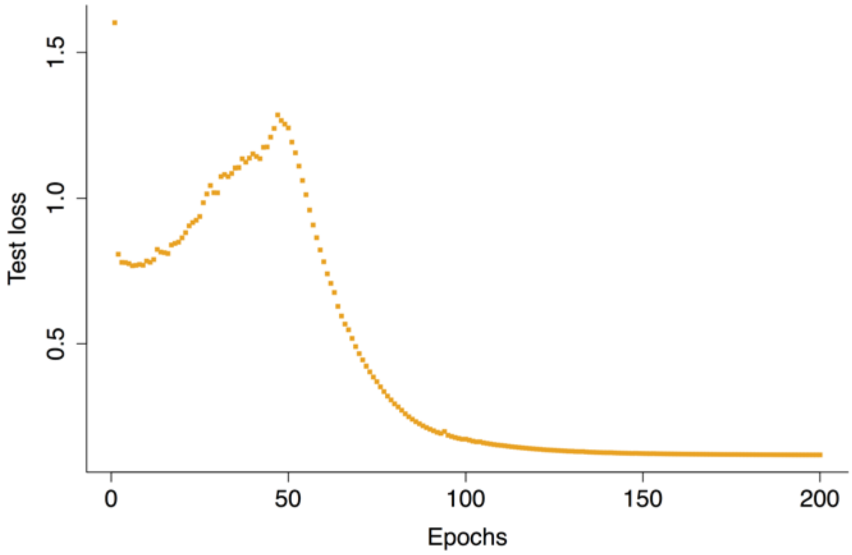

(B) Loss of testing data

Supplementary Figure S1: Loss of trianing data and testing data of the first fold (*LSTM with TI*)

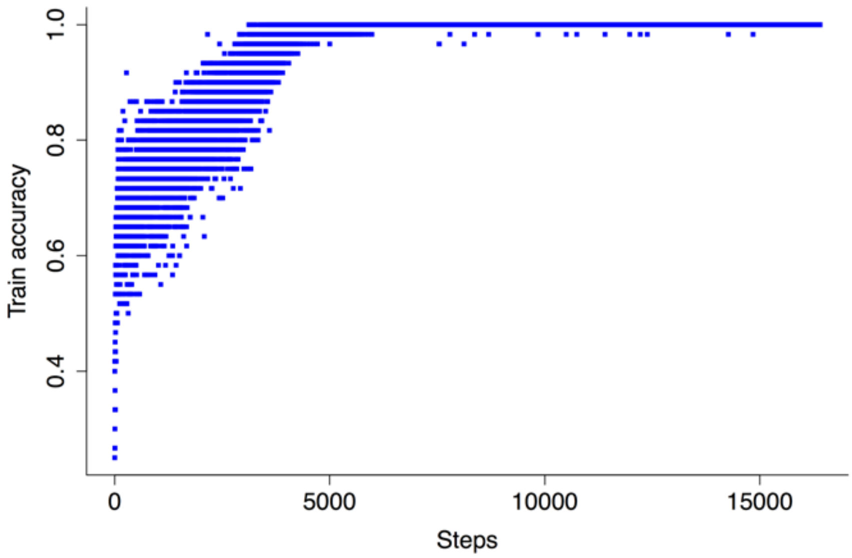

(A) Accuracy of training data

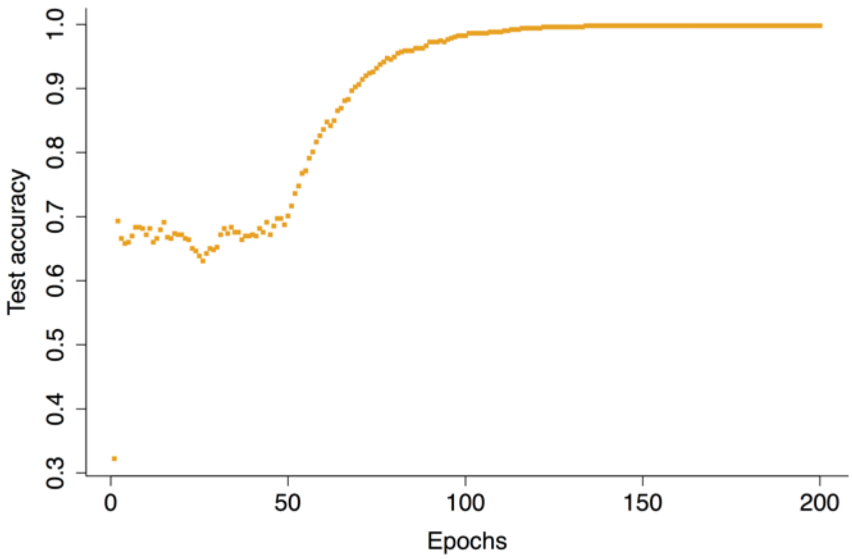

(B) Accuracy of testing data

Supplementary Figure S2: Accuracy of trianing data and test data of the first fold (*LSTM with TI*)

Accuracy, PPIA, SPIA in each fold for Table 3 and Table 4 in the main text

Accuracy, PPIA and SPIA in each fold of 10-fold cross validation for all models in this study are illustrated in the following tables (Supplementary Table S2 – Supplementary Table S14).

Supplementary Table S2. Values of Accuracy, PPIA and SPIA of 10-fold cross validation (*LSTM with TI*)

Folds	Accuracy	PPIA	SPIA
1st-Fold	0.9981	0.9947	0.9980
2nd-Fold	0.9922	0.9906	0.9922
3rd-Fold	0.9883	0.9847	0.9902
4th-Fold	0.9941	1.0000	0.9941
5th-Fold	0.9844	0.9803	0.9843
6th-Fold	0.9902	1.0000	0.9902
7th-Fold	0.9941	0.9901	0.9941
8th-Fold	0.9902	0.9791	0.9902
9th-Fold	0.9844	0.9900	0.9862
10th-Fold	0.9902	0.9843	0.9921
**Mean**	**0.9906**	**0.9894**	**0.9912**
**Standard Deviation**	**0.0043**	**0.0074**	**0.0039**

Supplementary Table S3. Values of Accuracy, PPIA and SPIA of 10-fold cross validation (*LSTM w/o TI*)

Folds	Accuracy	PPIA	SPIA
1st-Fold	0.9883	0.9788	0.9883
2nd-Fold	0.9804	0.9671	0.9805
3rd-Fold	0.9863	0.9796	0.9863
4th-Fold	0.9843	0.9781	0.9844
5th-Fold	0.9844	0.9803	0.9862
6th-Fold	0.9941	0.9948	0.9941
7th-Fold	0.9765	0.9803	0.9802
8th-Fold	0.9922	0.9948	0.9922
9th-Fold	0.9785	0.9851	0.9783
10th-Fold	0.9785	0.9529	0.9784
**Mean**	**0.9843**	**0.9792**	**0.9849**
**Standard Deviation**	**0.0057**	**0.0117**	**0.0053**

Supplementary Table S4. Values of Accuracy, PPIA and SPIA of 10-fold cross validation (LR)

Training methods	Training with average aggregation			Training with two previous visits			Training with one previous visits		
Folds	Accuracy	PPIA	SPIA	Accuracy	PPIA	SPIA	Accuracy	PPIA	SPIA
1st-Fold	0.8242	0.709	0.8242	0.6543	0.4762	0.6543	0.6758	0.4868	0.6758
2nd-Fold	0.8262	0.7418	0.8262	0.6543	0.5023	0.6543	0.6738	0.5164	0.6738

3rd-Fold	0.7793	0.7296	0.7808	0.6953	0.5867	0.6967	0.6777	0.5612	0.6791
4th-Fold	0.7891	0.7213	0.7891	0.6680	0.5082	0.6680	0.6699	0.4973	0.6699
5th-Fold	0.7676	0.6847	0.7736	0.6816	0.5271	0.6870	0.6855	0.5517	0.6909
6th-Fold	0.7949	0.7083	0.7965	0.6660	0.4896	0.6654	0.6855	0.5104	0.6849
7th-Fold	0.7891	0.7192	0.7984	0.6445	0.4975	0.6502	0.6602	0.4828	0.6660
8th-Fold	0.7656	0.6335	0.7671	0.6602	0.4293	0.6614	0.7090	0.5026	0.7104
9th-Fold	0.8047	0.7612	0.8107	0.6484	0.5373	0.6529	0.6387	0.5025	0.6430
10th-Fold	0.8145	0.7173	0.8193	0.6797	0.5026	0.6837	0.7266	0.5969	0.7308
**Mean**	**0.7955**	**0.7126**	**0.7986**	**0.6652**	**0.5057**	**0.6674**	**0.6803**	**0.5209**	**0.6825**
**Std. Dev.**	**0.0216**	**0.0345**	**0.0211**	**0.0162**	**0.0409**	**0.0163**	**0.0243**	**0.0370**	**0.0243**

Supplementary Table S5. Values of Accuracy, PPIA and SPIA of 10-fold cross validation (SVM)

Training methods	Training with average aggregation			Training with two previous visits			Training with one previous visits		
Folds	Accuracy	PPIA	SPIA	Accuracy	PPIA	SPIA	Accuracy	PPIA	SPIA
1st-Fold	0.7383	0.5873	0.7383	0.6387	0.4603	0.6387	0.6738	0.4656	0.6738
2nd-Fold	0.7773	0.6667	0.7773	0.6582	0.5352	0.6582	0.6699	0.4977	0.6699
3rd-Fold	0.7578	0.6939	0.7593	0.6641	0.5306	0.6654	0.6660	0.5204	0.6673
4th-Fold	0.7656	0.7049	0.7656	0.6719	0.4809	0.6719	0.6660	0.4754	0.6660
5th-Fold	0.7285	0.6749	0.7343	0.6699	0.5320	0.6732	0.6934	0.5222	0.6969
6th-Fold	0.7383	0.6094	0.7397	0.6504	0.4479	0.6497	0.6816	0.5052	0.6810
7th-Fold	0.7090	0.5862	0.7154	0.6523	0.5025	0.6601	0.6719	0.4828	0.6770
8th-Fold	0.7090	0.5864	0.7104	0.6387	0.3979	0.6399	0.6758	0.4555	0.6751
9th-Fold	0.7617	0.7164	0.7653	0.6211	0.4876	0.6252	0.6328	0.4826	0.6351
10th-Fold	0.7598	0.6387	0.7623	0.6680	0.4503	0.6699	0.7148	0.5236	0.7151
**Mean**	**0.7445**	**0.6465**	**0.7468**	**0.6533**	**0.4825**	**0.6552**	**0.6746**	**0.4931**	**0.6757**
**Std. Dev.**	**0.0237**	**0.0516**	**0.0226**	**0.0165**	**0.0445**	**0.0163**	**0.0209**	**0.0245**	**0.0208**

Supplementary Table S6. Values of Accuracy, PPIA and SPIA of 10-fold cross validation (DT)

Training methods	Training with average aggregation			Training with two previous visits			Training with one previous visits		
Folds	Accuracy	PPIA	SPIA	Accuracy	PPIA	SPIA	Accuracy	PPIA	SPIA
1st-Fold	0.7090	0.6296	0.7090	0.5976	0.4550	0.5977	0.6133	0.4815	0.6133
2nd-Fold	0.7383	0.6573	0.7383	0.5371	0.3615	0.5371	0.5625	0.3850	0.5625
3rd-Fold	0.6816	0.6327	0.6830	0.6035	0.4388	0.6047	0.5859	0.4745	0.5871
4th-Fold	0.7305	0.6120	0.7305	0.5820	0.3880	0.5820	0.6152	0.4973	0.6152
5th-Fold	0.6914	0.5961	0.6929	0.5781	0.4926	0.5827	0.5996	0.5123	0.6024

6th-Fold	0.7031	0.6146	0.7045	0.5957	0.4896	0.5969	0.5976	0.4531	0.5969
7th-Fold	0.6973	0.6749	0.7055	0.5625	0.4138	0.5672	0.5566	0.4680	0.5632
8th-Fold	0.6758	0.5916	0.6771	0.5918	0.4450	0.5930	0.5976	0.4031	0.5988
9th-Fold	0.6895	0.6070	0.6943	0.5723	0.5025	0.5759	0.6093	0.5224	0.6134
10th-Fold	0.7188	0.6073	0.7230	0.5898	0.4764	0.5914	0.5781	0.5079	0.5815
**Mean**	**0.7035**	**0.6223**	**0.7058**	**0.5810**	**0.4463**	**0.5829**	**0.5916**	**0.4705**	**0.5934**
**Std. Dev.**	**0.0206**	**0.0267**	**0.0200**	**0.0199**	**0.0470**	**0.0196**	**0.0204**	**0.0458**	**0.0196**

Supplementary Table S7. Values of Accuracy, PPIA and SPIA of 10-fold cross validation (RF)

Training methods	Training with average aggregation			Training with two previous visits			Training with one previous visits		
Folds	Accuracy	PPIA	SPIA	Accuracy	PPIA	SPIA	Accuracy	PPIA	SPIA
1st-Fold	0.7089	0.5714	0.7089	0.6230	0.3915	0.6230	0.6562	0.4339	0.6563
2nd-Fold	0.7051	0.5821	0.7051	0.6387	0.4272	0.6387	0.6328	0.4225	0.6328
3rd-Fold	0.6855	0.5612	0.6869	0.6250	0.4949	0.6262	0.6504	0.4847	0.6517
4th-Fold	0.7031	0.6393	0.7031	0.6641	0.4918	0.6641	0.6406	0.4372	0.6406
5th-Fold	0.6973	0.6010	0.7027	0.6426	0.4828	0.6476	0.6074	0.4532	0.6102
6th-Fold	0.6855	0.5573	0.6869	0.6387	0.4948	0.6399	0.6621	0.4792	0.6634
7th-Fold	0.6797	0.6010	0.6877	0.6250	0.4384	0.6324	0.6465	0.4631	0.6542
8th-Fold	0.6367	0.4712	0.6379	0.6250	0.3770	0.6262	0.6172	0.3770	0.6184
9th-Fold	0.7148	0.6418	0.7219	0.6191	0.4478	0.6252	0.6406	0.5274	0.6469
10th-Fold	0.6992	0.5602	0.7033	0.6719	0.4712	0.6758	0.6621	0.4921	0.6660
**Mean**	**0.6916**	**0.5786**	**0.6944**	**0.6373**	**0.4517**	**0.6399**	**0.6416**	**0.4570**	**0.6441**
**Std. Dev.**	**0.0223**	**0.0487**	**0.0227**	**0.0181**	**0.0430**	**0.0179**	**0.0183**	**0.0422**	**0.0186**

Supplementary Table S8. Values of Accuracy, PPIA and SPIA of 10-fold cross validation (Model without CDR)

Folds	Accuracy	PPIA	SPIA
1st-Fold	0.9512	0.9312	0.9512
2nd-Fold	0.9492	0.9437	0.9492
3rd-Fold	0.9531	0.9133	0.9530
4th-Fold	0.9648	0.9727	0.9648
5th-Fold	0.9668	0.9606	0.9685
6th-Fold	0.9512	0.9531	0.9511
7th-Fold	0.9609	0.9803	0.9664
8th-Fold	0.9395	0.9005	0.9393
9th-Fold	0.9414	0.9353	0.9428
10th-Fold	0.9785	0.9791	0.9784
**Mean**	**0.9557**	**0.9470**	**0.9565**

**Standard Deviation**	**0.0121**	**0.0273**	**0.0125**

Supplementary Table S9. Values of Accuracy, PPIA and SPIA of 10-fold cross validation (Model without GDS)

Folds	Accuracy	PPIA	SPIA
1st-Fold	0.9902	0.9894	0.9902
2nd-Fold	0.9883	0.9718	0.9883
3rd-Fold	0.9883	0.9718	0.9883
4th-Fold	0.9980	1.0000	0.9980
5th-Fold	0.9902	0.9803	0.9902
6th-Fold	0.9941	0.9896	0.9941
7th-Fold	0.9766	0.9704	0.9783
8th-Fold	0.9922	0.9791	0.9922
9th-Fold	0.9941	0.9950	0.9961
10th-Fold	0.9883	0.9843	0.9902
**Mean**	**0.9900**	**0.9832**	**0.9906**
**Standard Deviation**	**0.0057**	**0.0103**	**0.0054**

Supplementary Table S10. Values of Accuracy, PPIA and SPIA of 10-fold cross validation (Model without FAQ)

Folds	Accuracy	PPIA	SPIA
1st-Fold	0.9512	0.9153	0.9512
2nd-Fold	0.9551	0.9155	0.9551
3rd-Fold	0.9805	0.9694	0.9804
4th-Fold	0.9609	0.9617	0.9609
5th-Fold	0.9707	0.9606	0.9724
6th-Fold	0.9395	0.9062	0.9393
7th-Fold	0.9492	0.936	0.9526
8th-Fold	0.9551	0.9319	0.955
9th-Fold	0.9805	0.9851	0.9822
10th-Fold	0.9727	0.9476	0.9745
**Mean**	**0.9615**	**0.9429**	**0.9624**
**Standard Deviation**	**0.0140**	**0.0262**	**0.0143**

Supplementary Table S11. Values of Accuracy, PPIA and SPIA of 10-fold cross validation (Model without CDR, GDS)

Folds	Accuracy	PPIA	SPIA

1st-Fold	0.9805	0.9683	0.9805
2nd-Fold	0.9590	0.9296	0.9590
3rd-Fold	0.9785	0.9592	0.9785
4th-Fold	0.9707	0.9727	0.9707
5th-Fold	0.9727	0.9606	0.9744
6th-Fold	0.9629	0.9688	0.9628
7th-Fold	0.9531	0.9606	0.9565
8th-Fold	0.9668	0.9529	0.9667
9th-Fold	0.9746	0.9851	0.9763
10th-Fold	0.9805	0.9791	0.9823
**Mean**	**0.9699**	**0.9637**	**0.9708**
**Standard Deviation**	**0.0094**	**0.0154**	**0.0091**

Supplementary Table S12. Values of Accuracy, PPIA and SPIA of 10-fold cross validation (Model without CDR, FAQ)

Folds	Accuracy	PPIA	SPIA
1st-Fold	0.6699	0.6243	0.6699
2nd-Fold	0.7285	0.7183	0.7285
3rd-Fold	0.7109	0.6429	0.7123
4th-Fold	0.7539	0.7705	0.7539
5th-Fold	0.7285	0.7291	0.7323
6th-Fold	0.7402	0.7083	0.7417
7th-Fold	0.6836	0.6502	0.6897
8th-Fold	0.7324	0.6545	0.7319
9th-Fold	0.6953	0.6567	0.6963
10th-Fold	0.7480	0.7120	0.7505
**Mean**	**0.7191**	**0.6867**	**0.7207**
**Standard Deviation**	**0.0282**	**0.0471**	**0.0278**

Supplementary Table S13. Values of Accuracy, PPIA and SPIA of 10-fold cross validation (Model without GDS, FAQ)

Folds	Accuracy	PPIA	SPIA
1st-Fold	0.9590	0.9365	0.9590
2nd-Fold	0.9609	0.9343	0.9609
3rd-Fold	0.9824	0.9796	0.9824
4th-Fold	0.9824	0.9836	0.9824
5th-Fold	0.9766	0.9754	0.9764
6th-Fold	0.9531	0.9531	0.9530
7th-Fold	0.9648	0.9704	0.9704

8th-Fold	0.9961	1.0000	0.9961
9th-Fold	0.9570	0.9502	0.9586
10th-Fold	0.9570	0.9372	0.9587
**Mean**	**0.9689**	**0.9620**	**0.9698**
**Standard Deviation**	**0.0144**	**0.0229**	**0.0141**

Supplementary Table S14. Values of Accuracy, PPIA and SPIA of 10-fold cross validation (Model without CDR, GDS, FAQ)

Folds	Accuracy	PPIA	SPIA
1st-Fold	0.7031	0.6561	0.7031
2nd-Fold	0.7402	0.7371	0.7402
3rd-Fold	0.7461	0.7092	0.7456
4th-Fold	0.6699	0.6448	0.6699
5th-Fold	0.7207	0.6601	0.7244
6th-Fold	0.7305	0.7031	0.7319
7th-Fold	0.6973	0.67	0.6996
8th-Fold	0.7227	0.6754	0.7241
9th-Fold	0.7246	0.7015	0.7258
10th-Fold	0.6758	0.5759	0.6798
**Mean**	**0.7148**	**0.6868**	**0.7147**
**Standard Deviation**	**0.0355**	**0.0437**	**0.0353**

Supplementary Table S15. Mean and standard deviation of 10-fold cross validation (full model, basic model, and basic model with incorporating one feature of CDR/FAQ category at a time)

Category	Models	Accuracy	PPIA	SPIA
Full model	Model with basic information, CDR, FAQ, GDS	0.9906 ± 0.0043	0.9894 ± 0.0074	0.9912 ± 0.0039
Basic model	Model only with basic information (Model without CDR, FAQ, GDS)	0.7148 ± 0.0355	0.6868 ± 0.0437	0.7147 ± 0.0353
Basic model + one of CDR features	Basic model + MEMORY	0.8053 ± 0.0232	0.7869 ± 0.0474	0.8067 ± 0.0355
	Basic model + ORIENT	0.8254 ± 0.0413	0.8003 ± 0.0528	0.8271 ± 0.0475
	Basic model + JUDGMENT	0.8246 ± 0.0385	0.7969 ± 0.0568	0.8244 ± 0.0381
	Basic model + COMMUN	0.8258 ± 0.0371	0.8060 ± 0.0484	0.8266 ± 0.0448
	Basic model + HOMEHOBB	0.8293 ± 0.0399	0.8069 ± 0.0554	0.8253 ± 0.0446
	Basic model + PERSCARE	0.7965 ± 0.0446	0.7786 ± 0.0526	0.7967 ± 0.0454
	Basic model + CDRSUM	0.8188 ± 0.0401	0.8137 ± 0.0383	0.8173 ± 0.0385
	Basic model + CDRLANG	0.7373 ± 0.0428	0.7344 ± 0.0326	0.7294 ± 0.0409
	Basic model + COMPORT	0.7498 ± 0.0532	0.7340 ± 0.0494	0.7476 ± 0.0454
Basic model +	Basic model + BILLS	0.7924 ± 0.0189	0.7693 ± 0.0377	0.7826 ± 0.0255
	Basic model + TAXES	0.7877 ± 0.0278	0.7641 ± 0.0322	0.7875 ± 0.0326

one of FAQ features	Basic model + SHOPPING	0.7779 ± 0.0313	0.7797 ± 0.0438	0.7768 ± 0.0411
	Basic model + GAMES	0.7992 ± 0.0380	0.7951 ± 0.0380	0.7972 ± 0.0415
	Basic model + STOVE	0.7865 ± 0.0362	0.7858 ± 0.0255	0.7794 ± 0.0394
	Basic model + MEALPREP	0.7879 ± 0.0446	0.7749 ± 0.0573	0.7903 ± 0.0440
	Basic model + EVENTS	0.7459 ± 0.0307	0.7234 ± 0.0377	0.7468 ± 0.0358
	Basic model + PAYATTN	0.7645 ± 0.0247	0.7518 ± 0.0302	0.7695 ± 0.0235
	Basic model + REMDATES	0.7912 ± 0.0285	0.7885 ± 0.0468	0.7870 ± 0.0355
	Basic model + TRAVEL	0.7834 ± 0.0278	0.7782 ± 0.0392	0.7864 ± 0.0331
